# Integrated untargeted metabolomics and lipidomics reveals functional- and taste-related compounds in stir-fried *Trichosanthis Semen*

**DOI:** 10.3389/fnut.2026.1754969

**Published:** 2026-06-09

**Authors:** Yuan Wang, Di Wu, Xin Zhang, Xin Jia, Lijuan Ha

**Affiliations:** 1 The Second Affiliated Hospital of Heilongjiang University of Chinese Medicine, Harbin, China; 2 Department of Acupuncture and Massage, Changchun University of Chinese Medicine, Changchun, China

**Keywords:** lipidomics, liquid chromatography-high resolution mass spectrometry, metabolomics, molecular docking, *Trichosanthis Semen*

## Abstract

*Trichosanthis Semen* (TS) is widely used as both a food and traditional medicine. It is rich in diverse compounds (e.g., fatty acids, amino acids, and terpenes), which contribute to its functional and sensory properties. Stir-frying alters the functional and sensory properties of TS, yet the underlying chemical basis remains unclear. In this study, an integrated untargeted metabolomics and lipidomics strategy based on liquid chromatography-high resolution mass spectrometry (LC-HRMS) was employed to characterize compositional differences between raw and stir-fried TS. A total of 144 metabolites and 295 lipids were identified. Omics analysis revealed that 29 metabolites and 54 lipids exhibited significant changes after stir-frying, of which 25 compounds showed increased abundance. Based on these differential compounds, molecular docking suggested that 19 upregulated compounds may have potential relevance to constipation-related regulation, while sensory analysis tentatively linked 39 altered compounds to taste-related attributes. Overall, this study provides a comprehensive chemical profiling of TS and offers a preliminary link between processing-induced chemical changes and functional as well as sensory properties.

## 1 Introduction

*Trichosanthis Semen* (TS, *commonly known as “Diao Guazi” in traditional Chinese medicine*) is the dried mature fruit of *Trichosanthes kirilowii Maxim* or *Trichosanthes rosthornii Harms*, belonging to the family Cucurbitaceae (1). TS is rich in diverse compounds, including amino acids, fatty acids, lipids, and terpenoids (2). These chemical components are considered to contribute to the functional properties of TS, including lung-moistening, phlegm-resolving, and laxative-related effects (3–5). Food processing has been reported to alter the physicochemical properties of foods, thereby influencing their health benefits and sensory characteristics (6). Among various processing methods, stir-frying is widely used because it facilitates the retention of water-soluble nutrients while imparting distinct flavor and nutritional characteristics to foods (7, 8). Compared with raw TS, stir-fried TS is traditionally considered to exhibit reduced cold properties and enhanced intestine-moistening effects (9, 10). These processing-induced functional differences may be associated with alterations in chemical composition. However, limited studies have systematically investigated the compositional changes of TS after stir-frying. Therefore, it is necessary to comprehensively analyze the chemical compositions of raw and stir-fried TS.

Current methods for detecting compounds in TS include ultra-high performance liquid chromatography (UPLC), liquid chromatography-mass spectrometry (LC–MS), gas chromatography–mass spectrometry (GC–MS), and nuclear magnetic resonance (NMR) (11, 12). For example, Zhang et al. (10) identified 40, 41, and 38 components in extracts from TS fruit, peel, and seeds, respectively, using LC–MS. Similarly, Chu et al. (13) generated UPLC fingerprints of TS peel, seeds, and pulp revealing significant compositional differences among different parts of TS. Additionally, Yin et al. (14) identified 12 components from TS peel through extraction, isolation, and NMR analysis. However, limitations in sensitivity, coverage, and throughput restrict the comprehensive characterization of compounds in TS.

Liquid chromatography-high resolution mass spectrometry (LC-HRMS) offers high coverage, high throughput, and high sensitivity, and has been widely applied in the comprehensive analysis of complex samples (15). However, its application in TS remains limited. Untargeted metabolomics enables comprehensive metabolic profiling and facilitates the discovery of potential biomarkers (16). Nevertheless, metabolomics approaches generally focus on polar and moderately polar metabolites, with limited coverage of less polar lipids (17). In contrast, untargeted lipidomics enables comprehensive profiling of lipids in complex matrices (18). Therefore, the integration of LC-HRMS-based metabolomics and lipidomics may enable broader coverage for the characterization of compounds in TS.

This study aimed to systematically characterize the chemical compositions of raw and stir-fried TS and to explore the potential associations of differential compounds with functional and sensory properties. Comprehensive metabolite and lipid profiling of raw and stir-fried TS was performed using LC-HRMS. Subsequently, metabolomics and lipidomics analyses were conducted to screen significantly altered compounds between the two groups. Based on these differential compounds, molecular docking analysis was employed to explore their potential relevance to constipation-related targets. Finally, E-tongue analysis combined with correlation analysis was used to evaluate the potential associations between significantly altered compounds and taste attributes.

## 2 Materials and methods

### 2.1 Material

LC–MS grade acetonitrile (ACN), isopropanol, and ethanol were purchased from Merck (Darmstadt, Germany). LC–MS-grade formic acid and ammonium acetate were obtained from Macklin (Shanghai, China). Ultrapure water was prepared using a Milli-Q system (Millipore, Billerica, MA, USA).

### 2.2 Samples

**Figure 1:**
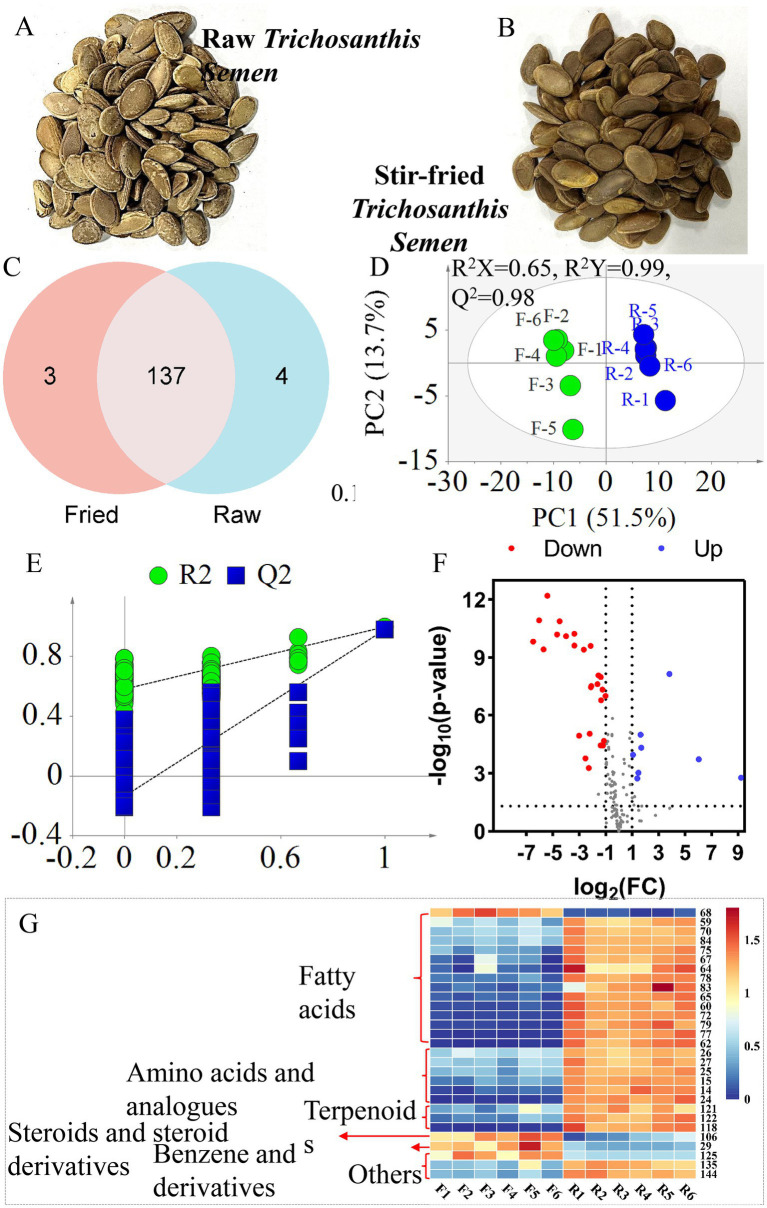
**(A)** Images of raw TS. **(B)** Images of stir-fried TS. **(C)** Venn plot. **(D)** PLS-DA score plot. **(E)** PLS-DA model permutation test. **(F)** Volcano plot. **(G)** Heatmap of 29 metabolites with significant changes.

TS samples used in this study were obtained from Fuzhou, Jiangxi, China. The stir-frying procedure was conducted according to the Chinese Pharmacopoeia and a previously reported method (51, 19). Briefly, 6 batches of cleaned TS were individually placed into a stir-frying pan and stir-fried at 80–100 °C until slight puffing and popping were observed. The stir-frying process lasted approximately 15 min, after which the samples were cooled to room temperature for subsequent analysis. Images of raw and stir-fried TS are shown in Figures 1A,B, respectively. Stir-frying was performed using an induction cooker, and the temperature was monitored using an infrared thermometer. Raw and stir-fried TS samples were subsequently ground and passed through a 40-mesh sieve to obtain homogeneous powders. The quality control (QC) sample was prepared by pooling equal amounts of raw and stir-fried TS powders.

The extraction procedures for metabolomics and lipidomics analyses were performed separately. For metabolite extraction, 0.6 g of TS powder was weighed into an Eppendorf tube and extracted with 3 mL of 80% (v/v) aqueous methanol. The mixture was ultrasonicated at 40 °C for 40 min. After centrifugation at 16,000 × g and 4 °C for 10 min, the supernatant was collected. For lipid extraction, 0.6 g of TS powder was treated with 3 mL of 80% (v/v) tert-butyl methyl ether/methanol solution, followed by ultrasonication at 30 °C for 15 min. After adding 1 mL of water and vortexing for 2 min, the mixture was centrifuged under the same conditions as described above. Both the metabolite and lipid extraction procedures were performed once for each sample.

### 2.3 LC-HRMS analysis

Metabolite and lipid separation was performed using a DI-AL6180 LC system (Hikvision, Hangzhou, China) coupled with a ZORBAX RRHD Eclipse Plus C18 column (2.1 × 100 mm, 1.8 μm; Agilent, USA). Data acquisition was carried out in both positive and negative ion modes using a ZenoTOF 7,600 mass spectrometer (AB SCIEX, USA). The injection volume was set to 5 μL, and the autosampler temperature was maintained at 6 °C.

For metabolomics analysis, the column temperature was set at 40 °C with a flow rate of 0.3 mL/min. In positive ion mode, mobile phase A consisted of water containing 0.1% formic acid, while mobile phase B was acetonitrile. The gradient program was as follows: 0–1 min, 20% B; 1–23 min, linear increase to 80% B; 23–27 min, held at 80% B; 27–27.1 min, returned to 20% B; 27.1–30 min, equilibration. In negative ion mode, mobile phase A consisted of water containing 5 mM ammonium acetate, and mobile phase B was ACN. The gradient program was as follows: 0–1 min, 20% B; 1–29 min, linear increase to 95% B; 29–32 min, held at 95% B; 32–32.1 min, returned to 20% B; 32.1–35 min, equilibration. For both ionization modes, the ion source temperature was set at 500 °C. The spray voltage and declustering potential were set at 5500 and 60 V, respectively. Gas 1 and gas 2 were both set as 50 psi. The scanning range for MS1 was 80–1,200 Da, while the MS/MS acquisition range was 50–1,200 Da. The collision-induced dissociation energy was set at 30 ± 15 eV. MS/MS acquisition was performed in information-dependent acquisition (IDA) mode, with 10 precursor ions selected per cycle.

For lipid analysis, the column temperature was set at 55 °C with a flow rate of 0.26 mL/min. In both positive and negative ion modes, mobile phase A consisted of acetonitrile/water (60:40, v/v), while mobile phase B consisted of isopropanol/acetonitrile (90:10, v/v). Both mobile phases contained 10 mM ammonium acetate. In positive ion mode, the gradient program was as follows: 0–1.5 min, 45% B; 1.5–15.5 min, linear increase to 85% B; 15.5–15.6 min, increased to 95% B; 15.6–18.0 min, held at 95% B; 18.0–18.1 min, returned to 45% B; 18.1–21.0 min, equilibration. In negative ion mode, the gradient program was as follows: 0–1.5 min, 30% B; 1.5–15.5 min, linear increase to 85% B; 15.5–15.6 min, increased to 90% B; 15.6–18.0 min, held at 90% B; 18.0–18.1 min, returned to 30% B; 18.1–21.0 min, equilibration. The mass spectrometry detection conditions for lipid analysis were identical to those used for metabolomic analysis. For both metabolomic and lipidomic analyses, one QC sample was injected after every four samples to evaluate system stability and analytical reproducibility.

### 2.4 Identification of metabolites and lipids

Metabolite and lipid identification in TS was performed with MS-DIAL software (version 5.5) (20). The databases used for metabolite identification included authentic standards integrated in MS-DIAL, MassBank, ReSpect, GNPS, Fiehn HILIC, MetaboBASE, RIKEN PlaSM, the Fiehn/Vaniya natural product library, and BMDMS-NP. The built-in lipid database in MS-DIAL was used for lipid identification. For both metabolite and lipid identification, the adduct forms in positive ion mode were set to [M + H] ^+^, [M + NH_4_] ^+^, [M + K] ^+^, [M + Na] ^+^, [M + H-H_2_O] ^+^, and [M + CH_3_OH + H] ^+^. The adduct forms in negative ion mode were set to [M-H] ^−^, [M-H_2_O-H] ^−^, and [M + CH_3_COO] ^−^. The relative and absolute MS/MS intensity cutoffs were set at 1% and 10, respectively. The thresholds for the dot product, weighted dot product, and reverse dot product score were set at 600, 500, and 600, respectively. The minimum number of matched MS/MS fragments was set to 3 for metabolite identification and 1 for lipid identification. All identification results were manually inspected to ensure accurate MS/MS matching and reasonable structural annotation of fragment ions.

### 2.5 Molecular docking

Previous studies have suggested that aquaporin 3 (AQP3) is associated with the pathophysiology of constipation (21). In addition, several studies have reported that modulation of AQP3 expression may contribute to constipation relief (22). Therefore, AQP3 was selected as the target protein for molecular docking. Ligand structures were obtained from the PubChem database^1^, while the crystal structure of the target protein was retrieved from the UniProt database^2^. Ligand structures were converted from sdf format to mol format using ChemDraw 3D 20.0 software. The docking conformation with the lowest binding energy (BE) was considered the most favorable binding conformation. Docking results were visualized using PyMOL software.

### 2.6 E-tongue analysis

The taste profile of TS was evaluated using a SuperTongue E-tongue system (ISENSO, USA) equipped with six sensors (S1-S6). Sample extraction followed the same procedure as that used for LC-HRMS-based metabolomic and lipidomic analyses, with the sample weight and solvent volume scaled up threefold. The extracts were collected, evaporated to dryness under a nitrogen stream, and reconstituted in 10 mL of deionized water prior to analysis. Each measurement lasted 120 s, during which signals were recorded at 1 s intervals. Continuous stirring at 60 r/min was applied to maintain sample uniformity, and a 30 s rinsing step was performed between runs to reduce carryover. Data processing was based on the mean signal obtained within the stable response window (100–120 s).

### 2.7 Data analysis

The Venn plot was created using the online platform Microbiome Bioinformatics^3^. The establishment and validation of the PLS-DA model were conducted with SIMCA-P 13 software (Umetrics, Umea, Sweden). Univariate analysis was performed using IBM SPSS Statistics 25.0 (International Business Machines Corporation, New York, NY, USA). Volcano plots were generated using GraphPad Prism (version 8.0). The heatmap was created using the online platform OmicStudio^4^. The Student’s t-test and Pearson’s correlation coefficients (*ρ*) were calculated using IBM SPSS 25.0 software (International Business Machines Corporation, New York).

## 3 Results and discussion

### 3.1 Identification of chemical constituents in TS

**Figure 2:**
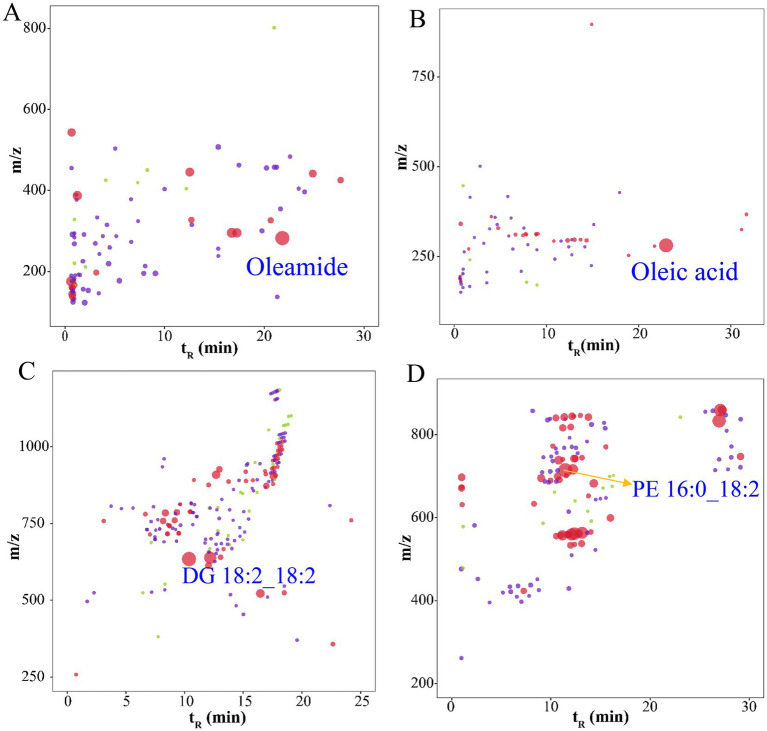
Distribution of t_R_ and *m/z* values of identified metabolites and lipids in positive and negative ion modes. **(A)** The positive ion modes of metabolites. **(B)** The negative ion modes of metabolites. **(C)** The positive ion modes of lipids. **(D)** The negative ion modes of lipids. Notably, the metabolite extraction may include some lipids, and the lipid extraction process may contain certain metabolites. The specific compounds are listed in Supplementary Tables S1, S2.

Based on MS-DIAL analysis, a total of 144 metabolites (Supplementary Table S1) and 295 lipids (Supplementary Table S2) were identified in TS. Metabolite distributions obtained in positive and negative ion modes are shown in Figures 2A,B, respectively. The results showed that the t_R_ of metabolites spanned nearly the entire elution gradient, indicating broad polarity coverage of metabolites in TS. In addition, most metabolites were eluted during the first half of the gradient, suggesting that the majority of compounds possessed moderate to high polarity. The m/z distribution of metabolites was mainly concentrated between 100 and 500 Da, indicating that most metabolites were of relatively low molecular weight. Among the identified metabolites, oleamide exhibited the highest relative abundance in positive ion mode, whereas oleic acid showed the highest relative abundance in negative ion mode. Previous studies have reported that oleamide is involved in the regulation of intestinal motility (23), while oleic acid may play a potential role in alleviating constipation-related symptoms (24). These findings suggest that the components rich in TS may be potentially associated with constipation-related physiological regulation. Nevertheless, the underlying mechanisms and biological relevance of these associations require further investigation. The t_R_ and m/z distribution of the identified lipids in positive and negative ion modes are shown in Figures 2C,D, respectively. Most lipids were eluted during the middle stage of the gradient, indicating that lipids in TS were predominantly low-polarity compounds. In addition, the m/z distribution of the lipids was mainly concentrated between 500 and 1,000 Da, indicating relatively high molecular weights for most lipids. Furthermore, DG 18:2_18:2 exhibited the highest average abundance in positive ion mode, whereas PE 16:0_18:2 showed the highest relative abundance in negative ion mode.

**Figure 3:**
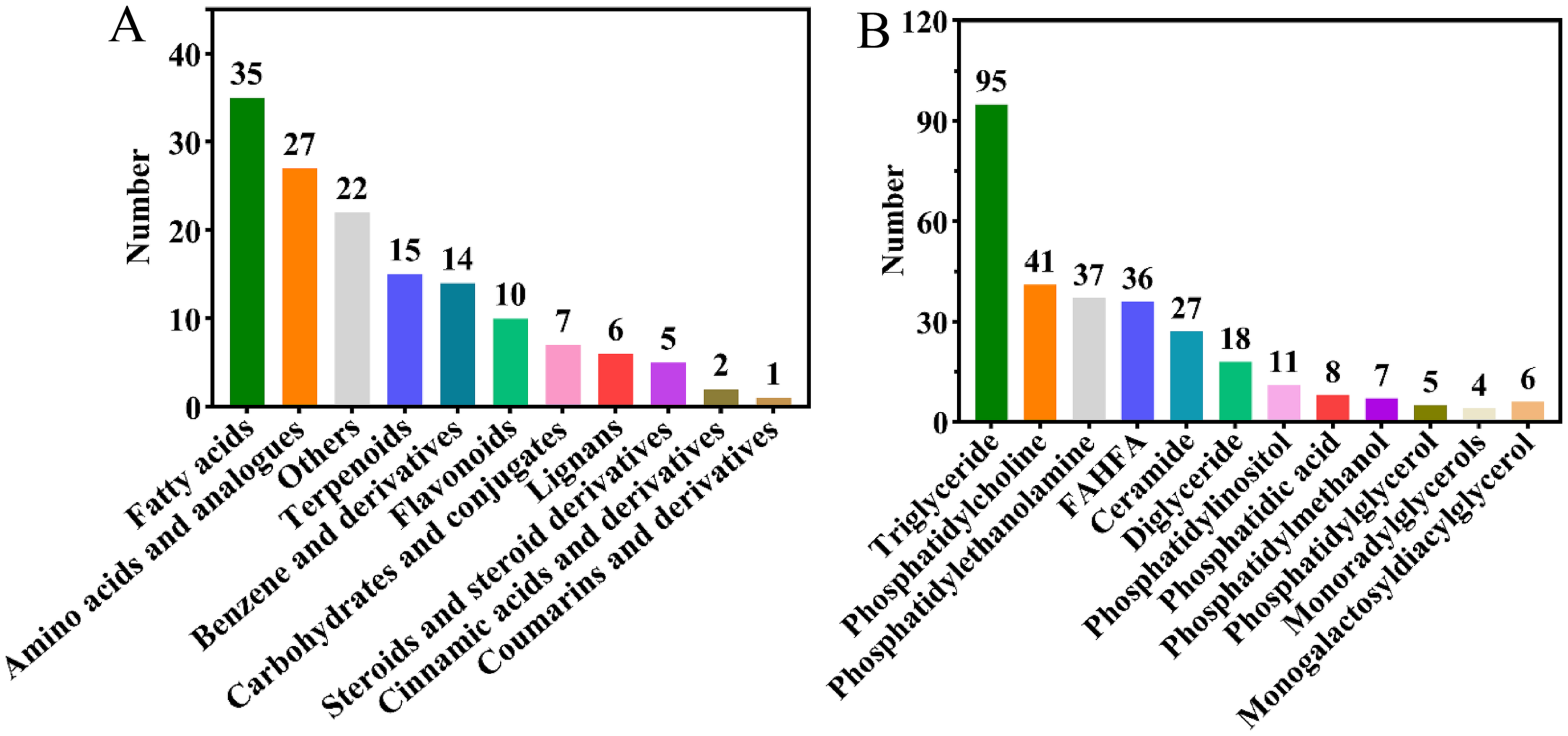
The classification of metabolites and lipids: **(A)** Metabolites and **(B)** lipids.

Figure 3A presents the classification of the 144 metabolites. As shown in the figure, the identified metabolites mainly consisted of 35 fatty acids, 27 amino acids and analogues, 15 terpenoids, 14 benzenes and derivatives, 10 flavonoids, 7 carbohydrates and conjugates, 6 lignans, 5 steroids and steroid derivatives, 2 cinnamic acids and derivatives, 1 coumarin and derivative, and 22 others. These results indicate that TS is rich in fatty acids and amino acids, which is in line with previous reports (25). Previous studies have reported that reduced levels of fecal fatty acids, such as acetic acid and propionic acid, may be associated with increased constipation severity (26). Moreover, alterations in amino acid metabolism have also been implicated in the development of constipation (27). The metabolite identification results therefore suggest a potential association between the chemical composition of TS and constipation-related physiological regulation. Figure 3B presents the classification of 295 lipids. Among these lipids, 95 triglycerides (TGs), 41 phosphatidylcholines (PCs), 37 phosphatidylethanolamines (PEs), 36 fatty acid esters of hydroxy fatty acids (FAHFAs), 27 ceramides (Cer_AP), 18 diglycerides (DGs), 11 phosphatidylinositols (PIs), 8 phosphatidic acids (PAs), 7 phosphatidylmethanols (PMeOHs), 6 monogalactosyldiacylglycerols (MGDGs), 5 phosphatidylglycerols (PGs), and 4 monoradylglycerols (MGs). These results indicate that TS is rich in TGs, PCs, PEs, and FAHFAs. Previous studies have suggested that PCs are important components of the intestinal mucus barrier and may contribute to gastrointestinal physiological regulation and mucosal protection (28). In addition, PEs have been implicated in intestinal metabolic homeostasis and gut microbiota regulation, suggesting their potential relevance to constipation-related physiological processes (29). Therefore, lipid identification results suggest that the lipid components enriched in TS may be potentially associated with its functional characteristics.

**Figure 4:**
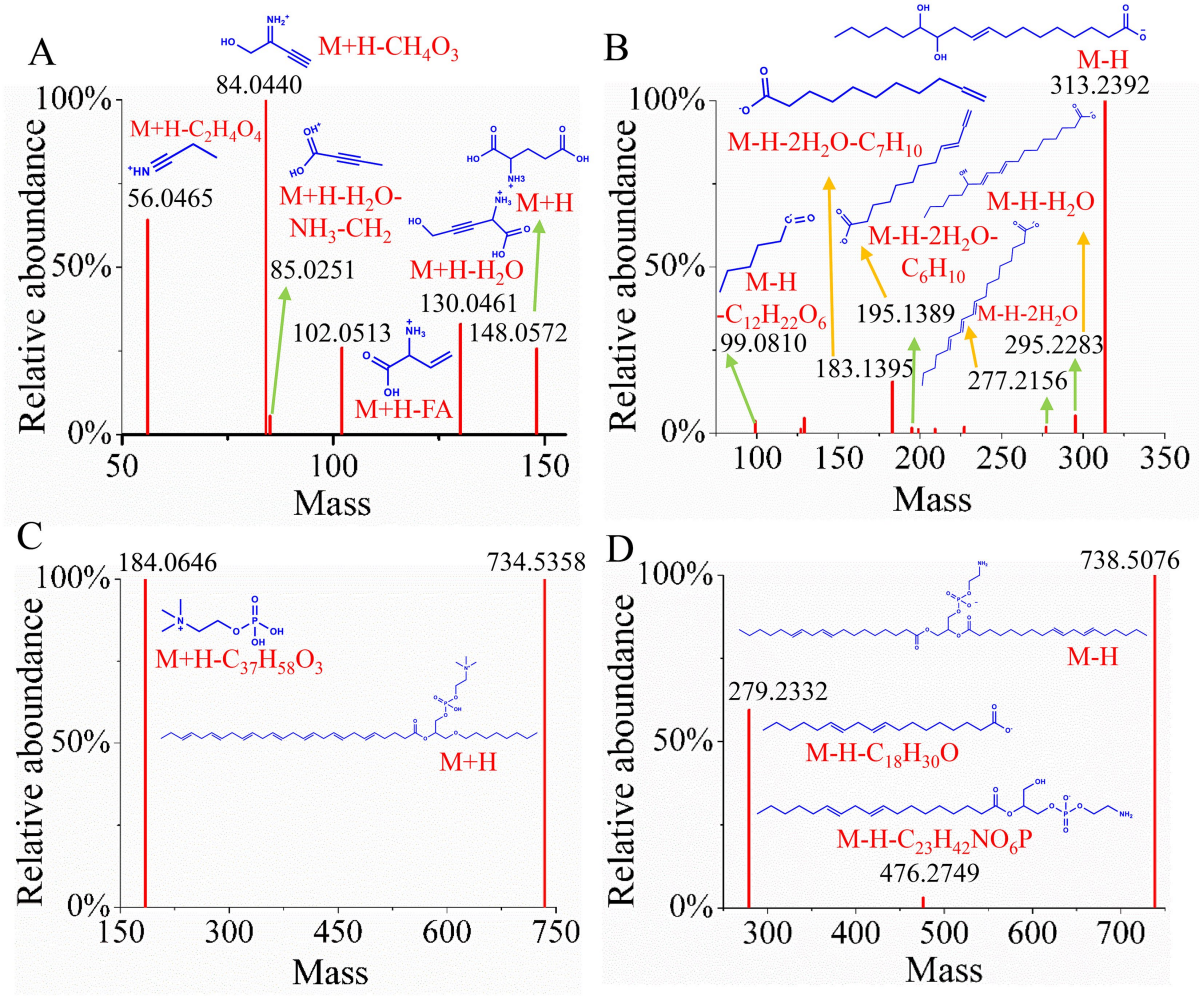
The fragment structure and MS/MS of compounds. **(A)** Glutamic acid. **(B)** 12,13-Dihydroxyoctadec-9-enoate. **(C)** PC O-8:0/26:7. **(D)** PE 36:4 | PE 18:2_18:2.

Following database-assisted identification of metabolites and lipids, manual verification was performed to improve the reliability of compound identification. The verification procedure was exemplified using four representative compounds. As shown in Figure 4A, the peak detected at t_R_ = 0.77 min and m/z = 148.0572 was preliminarily identified as glutamic acid based on database matching. Subsequently, the corresponding MS/MS fragmentation patterns were manually analyzed to validate the result. The ion at m/z 148.0572 corresponds to the quasi-molecular ion [M + H] ^+^. The fragment ion at m/z 130.0461 was generated by the loss of one H_2_O from the quasi-molecular ion, while the fragment at m/z 102.0513 arises from the loss of one formic acid. In addition, the fragment at m/z 85.0251 was produced through the sequential losses of H_2_O, NH_3_, and CH_2_. The fragments at m/z 84.0440 and 56.0465 correspond to losses of CH_4_O_3_ and C_2_H_4_O_4_, respectively. These fragmentation behaviors were consistent with the characteristic MS/MS fragmentation patterns of glutamic acid. Therefore, the compound corresponding to this peak was identified as glutamic acid.

As shown in Figure 4B, the peak at t_R_ of 9.09 min and m/z 313.2392 was preliminarily identified as 12,13-dihydroxyoctadec-9-enoate based on database matching. The ion at m/z 313.2392 corresponded to the quasi-molecular ion peak [M-H]^−^. The fragment with m/z 295.2283 was generated by the loss of one H_2_O from the quasi-molecular ion, while the fragment at m/z 277.2156 corresponded to the loss of two H_2_O. The fragment at m/z 195.1389 was formed from the loss of two H_2_O molecules and a C_6_H_10_, whereas the fragment at m/z 183.1395 resulted from the loss of two H_2_O molecules and a C_7_H_10_. In addition, the fragment at m/z 99.0810 was generated after the loss of C_12_H_22_O_6_. These fragmentation patterns were consistent with the characteristic MS/MS cleavage behavior of 12,13-dihydroxyoctadec-9-enoate. Therefore, the compound corresponding to this peak was identified as 12,13-dihydroxyoctadec-9-enoate.

As shown in Figure 4C, the MS/MS of PC O-8:0/26:7 exhibited a quasi-molecular ion M + H at m/z = 734.5358 and a characteristic fragment ion at m/z 184.0646, corresponding to the phosphocholine head group after dissociation of the fatty acyl chains. Similarly, as shown in Figure 4D, the MS/MS of PE 36:4|PE 18:2_18:2 displayed a quasi-molecular ion M-H at m/z = 738.5076 and a fragment ion at m/z 476.2749, corresponding to the loss of one fatty acyl chain. In addition, the fragment ion at m/z 279.2332 was assigned to the fatty acyl chain fragment.

### 3.2 Metabolomics of TS with stir-frying treatment

As shown in Figure 1C, 141 metabolites were detected in raw TS, whereas 140 metabolites were identified in stir-fried TS. Notably, 137 metabolites were shared between the two sample groups, suggesting a high degree of similarity in metabolite composition. These results indicate that stir-frying mainly affected metabolite abundance rather than overall metabolite composition. Notably, 3,9,15-tribenzyl-6,12,18-triisopropyl-4,10,16-trimethyl-1,7,13-trioxa-4,10,16-triazacyclooctadecane-2,5,8,11,14,17-hexone, FA 18:4 + 2O, jasmonic acid, and linolenic acid were exclusively detected in raw TS. Among these compounds, 3,9,15-tribenzyl-6,12,18-triisopropyl-4,10,16-trimethyl-1,7,13-trioxa-4,10,16-triazacyclooctadecane-2,5,8,11,14,17-hexone contains both peptide and ester bonds. Its disappearance after stir-frying may be attributed to thermal cleavage of these labile bonds during heating (30). The remaining three compounds uniquely detected in raw TS were fatty acid-related compounds. Their disappearance may be associated with thermal oxidation of unsaturated bonds or condensation reactions involving carboxyl groups during stir-frying (31). Conversely, N-methyl-N-[(2E,4E,6E)-2,4,6-octatrienoyl]valylalanylprolinamide, (Z)-2-octylpent-2-enedioic acid, and decanoic acid were exclusively detected in stir-fried TS. N-methyl-N-[(2E,4E,6E)-2,4,6-octatrienoyl]valylalanylprolinamide is a small peptide, and its formation may be associated with condensation reactions between amino acids and fatty acid-derived compounds during heating (32). In addition, (Z)-2-octylpent-2-enedioic acid and decanoic acid are fatty acid-related compounds that may originate from the thermal degradation of lipids such as glycerides and FAHFAs during stir-frying (33).

Figure 1D presents the PLS-DA score plot for metabolomic profiling. The values of R^2^X, R^2^Y, and Q^2^ were 0.65, 0.99, and 0.98, respectively, indicating good model performance. A clear separation between raw and stir-fried TS samples was observed, suggesting significant differences in their metabolite profiles. Combined with the results shown in Figure 1C, these findings indicate that stir-frying mainly affected metabolite abundance rather than metabolite composition. To further evaluate the robustness of the PLS-DA model, a permutation test was performed (Figure 1E). The permutation test yielded intercept values of R^2^ = 0.266 and Q^2^ = − 0.14, both of which were lower than those of the original model, indicating that the model was not overfitted. Figure 1F shows the volcano plot of differential metabolites screened using the criteria of *p* < 0.05 and fold change (FC) > 2 or < 0.5 between stir-fried and raw TS. After stir-frying, 26 metabolites showed decreased abundance, whereas 8 metabolites exhibited increased abundance. To further identify significantly altered metabolites, compounds with VIP > 1.2 were selected, as summarized in Figure 1G and Supplementary Table S3. Among the 29 significantly changed metabolites, 25 were downregulated after stir-frying. Notably, 15 fatty acids exhibited significant changes, among which 14 showed decreased abundance. In addition, the abundances of 6 amino acids and 3 terpenoid compounds were reduced after stir-frying.

In the metabolomic analysis, most significantly altered metabolites, particularly fatty acids and amino acids, exhibited reduced abundance after stir-frying. Unsaturated fatty acids are highly susceptible to thermal oxidation and degradation during heating, leading to the formation of smaller oxidation products or volatile compounds (34). Similarly, the decrease in amino acid abundance may be associated with Maillard reactions occurring during stir-frying, in which amino acids react with reducing sugars or carbonyl compounds under elevated temperatures to generate new reaction products (35). Meanwhile, several newly detected compounds in stir-fried TS, including peptide- and fatty acid-related compounds, further support the occurrence of thermal degradation, condensation, and lipid transformation reactions during processing.

### 3.3 Lipidomics of TS with stir-frying treatment

**Figure 5:**
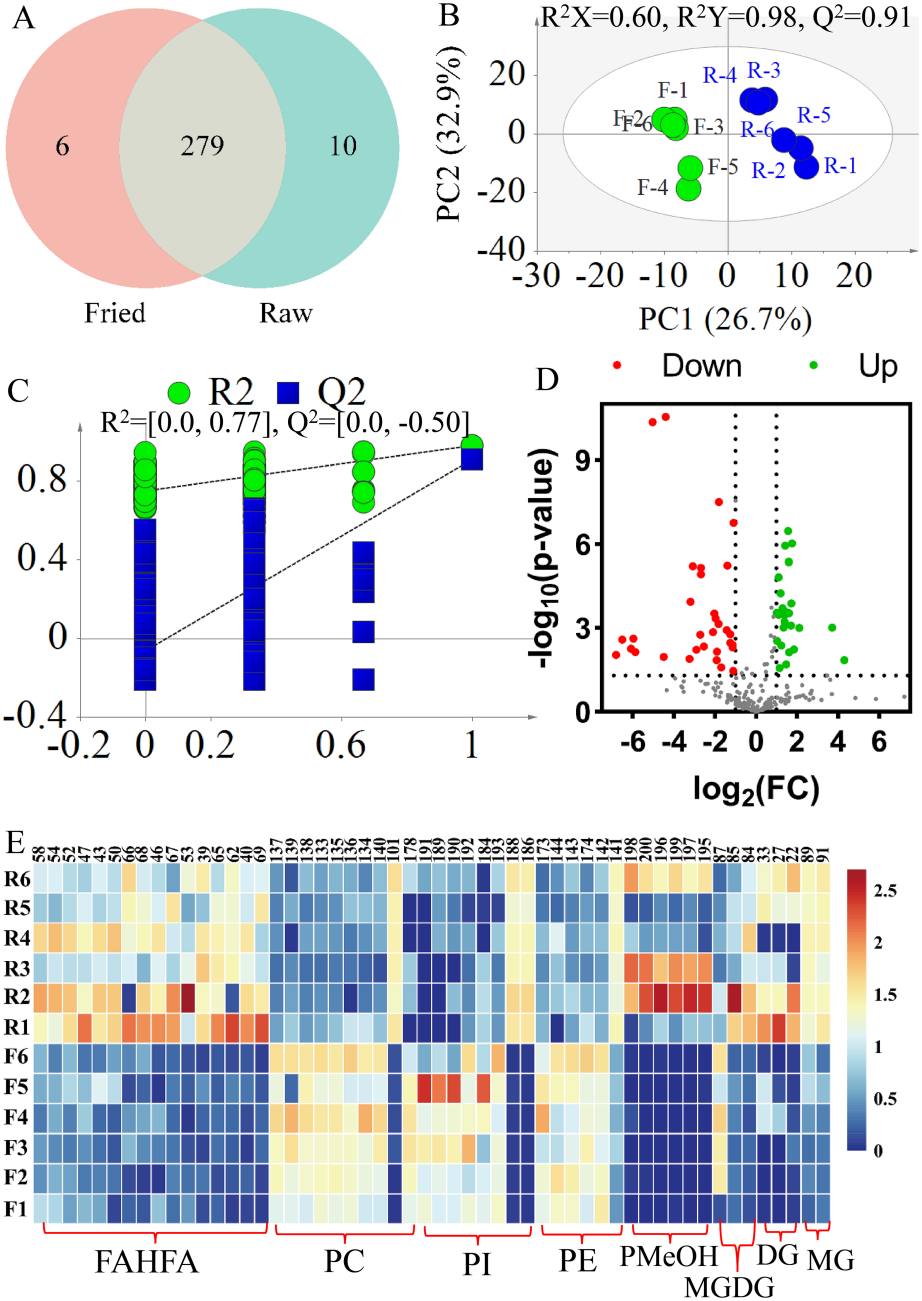
Statistical analysis results of lipids. **(A)** Venn plot. **(B)** PLS-DA score plot. **(C)** PLS-DA model permutation test. **(D)** Volcano plot. **(E)** Heatmap of 54 lipids with significant changes.

Figure 5A presents a Venn diagram illustrating the distribution of detected lipids in raw and stir-fried TS. As shown in Figure 5A, 285 lipids were identified in raw TS, whereas 289 lipids were detected in stir-fried TS, with 279 lipids shared between the two groups. Among the lipids detected in raw TS, 2 DGs, 2 FAHFAs, 1 Ceramide, 1 PC, and 4 TGs were no longer detected after stir-frying, while 1 ceramide, 1 PC, 3 PEs, and 1 TG were newly detected. Combined with the metabolomic results shown in Figure 1C, these findings suggest that stir-frying may not only reduce certain lipid species but also promote the formation of new lipids. These findings are consistent with previous reports on processing-induced lipid transformation during thermal treatment (15).

Figure 5B presents the PLS-DA plot comparing the lipids profiles of raw and stir-fried TS. The model exhibited R^2^X, R^2^Y, and Q^2^ values of 0.60, 0.98, and 0.91, respectively, indicating good predictive ability and model stability. In addition, a clear separation between raw and stir-fried TS samples was observed, suggesting significant differences in lipid composition between the two groups. Combined with the results shown in Figure 5A, these differences were mainly reflected in lipid abundance rather than lipid diversity. To further evaluate the robustness of the PLS-DA model, a permutation test was conducted (Figure 5C). The permutation test generated intercept values of R^2^ = 0.77 and Q^2^ = −0.50, both of which were lower than those of the original model, indicating that the model was not overfitted. Figure 5D shows the volcano plot of differential lipids screened using the criteria of *p* < 0.05 and FC > 2 or < 0.5 between stir-fried and raw TS. After stir-frying, 55 lipids showed decreased abundance, whereas 21 lipids exhibited increased abundance. To further identify significantly altered lipids associated with stir-frying, compounds with both VIP > 1.2 and significant changes in the volcano plot were selected (Figure 5E; Supplementary Table S4). Among the 54 significantly altered lipids, 33 showed decreased abundance after stir-frying. Notably, among the 10 significantly altered PCs, 9 exhibited increased abundance. Similarly, 6 of the 8 altered PIs and 5 of the 6 altered PEs were upregulated after stir-frying. In addition, 1 of the 3 significantly altered MGDGs showed increased abundance. In contrast, all significantly altered FAHFAs (16), PMeOHs (6), DGs (3), and MGs (2) showed decreased abundance after stir-frying.

The lipidomic results revealed a trend similar to that observed in metabolomics, with most significantly altered lipids showing decreased abundance after stir-frying. Among these lipids, FAHFAs represented the largest proportion of downregulated compounds, suggesting that these structurally labile lipids may be particularly sensitive to thermal treatment. Previous studies have shown that FAHFAs can undergo oxidation, hydrolysis, and structural rearrangement during heating (36, 37), which may explain their marked reduction after stir-frying. In contrast, several phospholipid subclasses, particularly PCs, PIs, and PEs, exhibited increased abundance after processing. Since these lipids contain fatty acyl chains and play important roles in membrane structure and lipid metabolism, their increased abundance may be associated with lipid remodeling and reacylation processes involving degradation-derived fatty acids generated during thermal treatment (52). Collectively, these findings indicate that stir-frying not only induces degradation of endogenous lipids, but may also promote the redistribution and reconstruction of lipid species in TS.

According to traditional processing theory and previous studies, stir-frying can alter the physicochemical properties and sensory characteristics of TS, thereby potentially affecting its functional properties and flavor profile. In addition, stir-frying is traditionally considered to reduce the “cold nature” of TS and improve its sensory acceptability (38). Because the functional and sensory characteristics of food materials are closely associated with their chemical composition, the compounds significantly altered after stir-frying may contribute to the processing-induced changes observed in TS. Several fatty acids markedly decreased after stir-frying, which may influence both the physiological and sensory properties of the processed product (39). Meanwhile, the increased abundance of phospholipid subclasses such as PCs, PIs, and PEs may also be associated with gastrointestinal physiological regulation, as these lipids have been reported to participate in intestinal barrier maintenance and gut homeostasis (40–42).

Taken together, these results suggest that the significant compositional variations induced by stir-frying may be potentially associated with both the functional characteristics and taste properties of TS. Therefore, further analyses were conducted to explore the potential relevance of significantly altered compounds to constipation-related mechanisms as well as their correlations with taste attributes.

### 3.4 Functional relevance of compounds with significant change: implication for constipation

**Figure 6:**
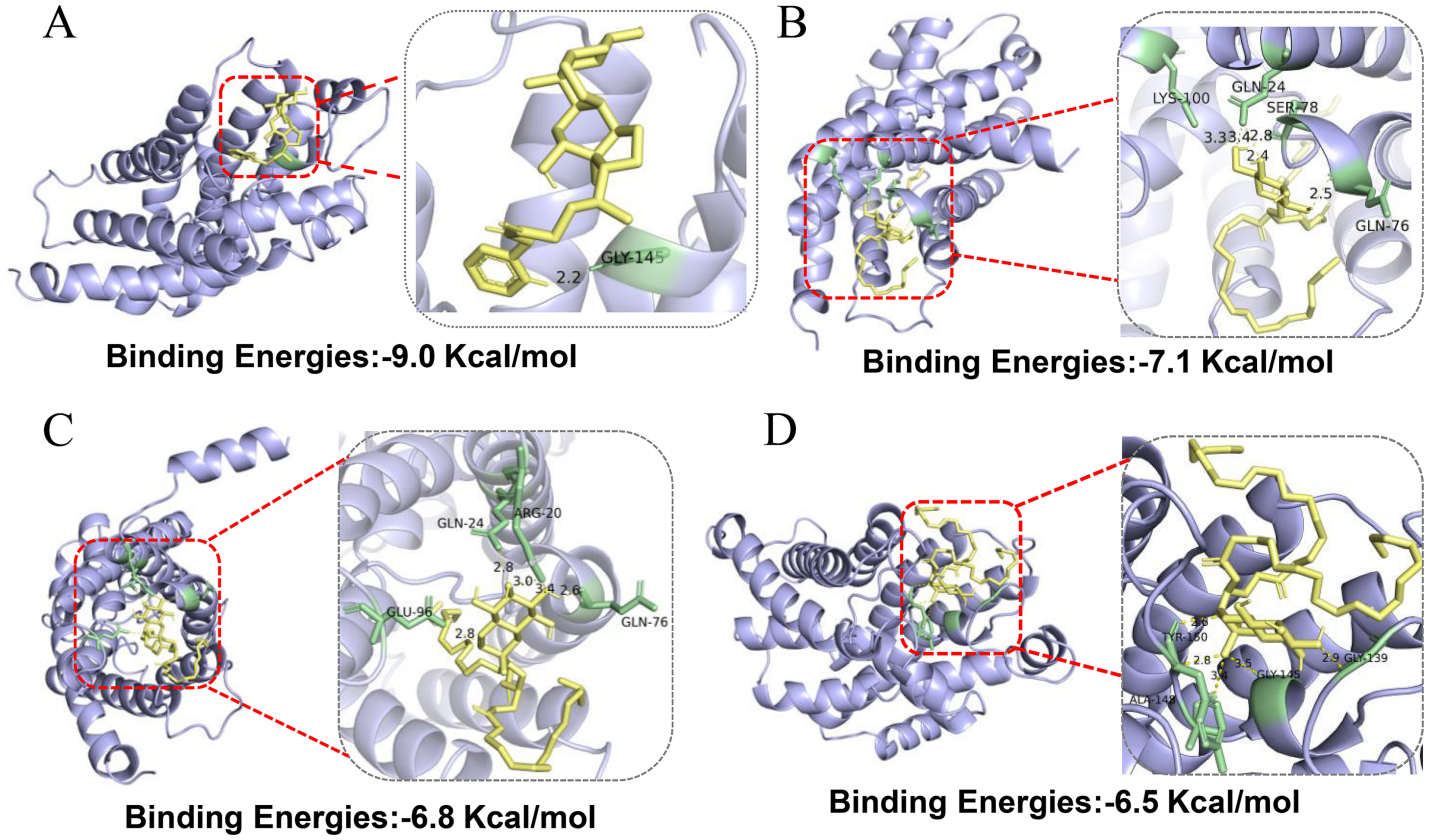
The 3D plot of molecular docking. **(A)** 4-HNP. **(B)** MGDG 36:4|MGDG 18:2_18:2. **(C)** PI 34:0. **(D)** PI 36:4|PI 18:2_18:2.

To investigate the potential relevance of significantly increased compounds to constipation-related regulation, 25 compounds with increased abundance were selected as ligands, while AQP3 was used as the target protein for molecular docking. As shown in Table 1, among the 25 compounds, 19 exhibited BE ≤ − 5.0 kcal/mol. Among these 19 compounds, 6 were identified as PIs, 5 as PCs, 4 as PEs, 1 as a PG, 1 as a steroid and steroid derivative, 1 as a benzene derivative, and 1 as an MG. Figure 6 shows the 3D interaction diagrams of compounds exhibiting BEs lower than −6.5 kcal/mol with AQP3. As shown in Figure 6A, (R)-4-(3,12-dihydroxy-10,13-dimethylhexadecahydro-1H-cyclopenta[A]phenanthren-17-yl)-N-(pyridin-2-ylmethyl) pentanamide (4-HNP) formed one hydrogen bond with GLY-145 and exhibited a BE of −9.0 kcal/mol. MGDG 36:4|MGDG 18:2_18:2 showed a BE of −7.1 kcal/mol with AQP3 and formed four hydrogen bonds with LYS-100, GLN-24, SER-78, and GLN-76 (Figure 6B). Similarly, PI 34:0 exhibited a BE of −6.8 kcal/mol and formed four hydrogen bonds were formed with ARG-20, GLN-24, GLU-96, and GLN-76 (Figure 6C). PI 36:4|PI 18:2_18:2 also demonstrated favorable binding to AQP3, with a BE of −6.5 kcal/mol and four hydrogen bonds formed with TYR-150, ALA-148, GLY-145, and GLY-139 (Figure 6D).

**Table 1:** The BE between compounds and the AQP3 protein.

t_R_/min	m/z	Name	Formula	MW^*^	Class	FC	*p*	VIP	BE (kcal/mol)
12.3	844.6002	PC 36:2|PC 18:0_18:2	C44H84NO8P	785.5935	Phosphatidylcholine	3.1	7.5E-03	1.5	−4.4
11.2	816.5737	PC 34:2|PC 16:0_18:2	C42H80NO8P	757.5622	Phosphatidylcholine	2.5	9.8E-04	1.7	−4.5
9.0	171.1386	Decanoic Acid	C10H20O2	172.1463	Fatty acids	14.1	7.2E-09	1.4	−4.6
20.6	326.2967	Oleoyl Ethanolamide	C20H39NO2	325.2981	Others	2.1	1.1E-04	1.2	−4.6
12.1	844.6045	PC 36:2|PC 18:1_18:1	C44H84NO8P	785.5935	Phosphatidylcholine	3.0	4.3E-06	1.9	−4.6
11.3	768.5490	DMPE 36:3|DMPE 18:1_18:2	C43H80NO8P	769.5622	Phosphatidylethanolamine	2.3	5.8E-05	1.9	−4.9
12.1	770.5651	DMPE 36:2|DMPE 18:1_18:1	C43H82NO8P	771.5778	Phosphatidylethanolamine	2.3	4.2E-03	1.6	−5.0
10.5	840.5737	PC 36:4|PC 18:2_18:2	C44H80NO8P	781.5622	Phosphatidylcholine	3.0	3.4E-07	2.0	−5.1
12.3	742.5367	PE 36:2|PE 18:1_18:1	C41H78NO8P	743.5465	Phosphatidylethanolamine	2.7	2.6E-04	1.8	−5.3
12.4	742.5346	PE 36:2|PE 18:0_18:2	C41H78NO8P	743.5465	Phosphatidylethanolamine	2.1	3.4E-04	1.8	−5.3
27.7	809.5151	PI 32:0	C41H79O13P	810.5258	Phosphatidylinositol	3.6	5.8E-03	1.5	−5.5
9.7	835.5306	PI 34:1|PI 16:0_18:1	C43H81O13P	836.5415	Phosphatidylinositol	2.6	3.5E-04	1.8	−5.5
13.0	846.6192	PC 36:1|PC 18:0_18:1	C44H86NO8P	787.6091	Phosphatidylcholine	2.4	2.0E-04	1.8	−5.6
11.3	842.5895	PC 36:3|PC 18:1_18:2	C44H82NO8P	783.5778	Phosphatidylcholine	2.7	1.1E-06	2.0	−5.7
27.6	847.5308	PI 35:2	C44H81O13P	848.5415	Phosphatidylinositol	4.3	1.0E-03	1.7	−5.7
12.0	818.5891	PC 34:1|PC 16:0_18:1	C42H82NO8P	759.5778	Phosphatidylcholine	3.4	9.5E-07	2.0	−5.9
28.2	771.5117	PG 36:3	C42H77O10P	772.5254	Phosphatidylglycerol	3.3	8.2E-04	1.7	−5.9
25.6	855.5001	PI 36:5	C45H77O13P	856.5102	Phosphatidylinositol	2.8	2.0E-02	1.4	−6.2
11.9	792.5739	PC 32:0|PC 16:0_16:0	C40H80NO8P	733.5622	Phosphatidylcholine	2.6	6.0E-04	1.7	−6.3
10.5	766.5344	DMPE 36:4|DMPE 18:2_18:2	C43H78NO8P	767.5465	Phosphatidylethanolamine	2.1	1.5E-05	1.9	−6.3
7.9	179.0349	4-Hydroxy-Phenyl-Pyruvic Acid	C9H8O4	180.0423	Benzene and derivatives	3.1	1.0E-05	1.3	−6.4
8.2	857.5129	PI 36:4|PI 18:2_18:2	C45H79O13P	858.5258	Phosphatidylinositol	3.3	1.3E-04	1.8	−6.5
29.1	837.5468	PI 34:0	C43H83O13P	838.5571	Phosphatidylinositol	13.1	9.7E-04	1.7	−6.8
11.4	837.5685	MGDG 36:4|MGDG 18:2_18:2	C45H78O10	778.5595	Monogalactosyldiacylglycerol	2.2	2.7E-02	1.3	−7.1
22.6	483.3448	4-HNP	C30H46N2O3	482.3508	Steroids and derivatives	3.2	4.7E-05	1.3	−9.0

^*^MW represents Molecular Weight.

The molecular docking results suggested that several compounds significantly upregulated after stir-frying may potentially interact with AQP3, a membrane channel protein closely associated with intestinal water transport and constipation-related physiological regulation. Previous studies have demonstrated that abnormal AQP3 expression is associated with impaired intestinal water balance and constipation symptoms, and modulation of AQP3 has therefore been considered a potential strategy for regulating gastrointestinal function (21, 22). In the present study, 19 of the 25 significantly upregulated compounds exhibited BE ≤ − 5.0 kcal/mol toward AQP3, indicating favorable binding affinity between these compounds and the target protein.

Among the compounds with relatively strong binding affinity, 4-HNP exhibited the lowest BE (−9.0 kcal/mol), suggesting a stable interaction with AQP3. Structurally, 4-HNP contains a steroid-like scaffold, previous studies have reported reduced steroid hormone levels in women with severe idiopathic constipation (43). In addition, MGDG 36:4|MGDG 18:2_18:2 also showed favorable binding affinity toward AQP3 through multiple hydrogen-bond interactions. Previous studies have suggested that MGDG-related galactolipids may participate in intestinal lipid metabolism and gut physiological regulation (44). Similarly, PI 34:0 and PI 36:4|PI 18:2_18:2 demonstrated relatively strong interactions with AQP3. PIs are known to participate in membrane signaling and intestinal physiological regulation (45), which may further support the potential relevance of these compounds to constipation-related processes.

Nevertheless, it should be noted that molecular docking only provides preliminary in silico evidence regarding potential ligand-protein interactions and cannot directly confirm biological activity or therapeutic efficacy. Moreover, constipation is a complex physiological process involving intestinal motility, water transport, gut microbiota, and neuroendocrine regulation. Therefore, the functional relevance of these significantly altered compounds requires further experimental validation, particularly through *in vivo* and mechanistic studies. Despite these limitations, the docking results provide preliminary insight into the potential association between processing-induced compositional changes and constipation-related physiological regulation in TS.

### 3.5 Correlation analysis between compounds with significant change and tastes

**Figure 7:**
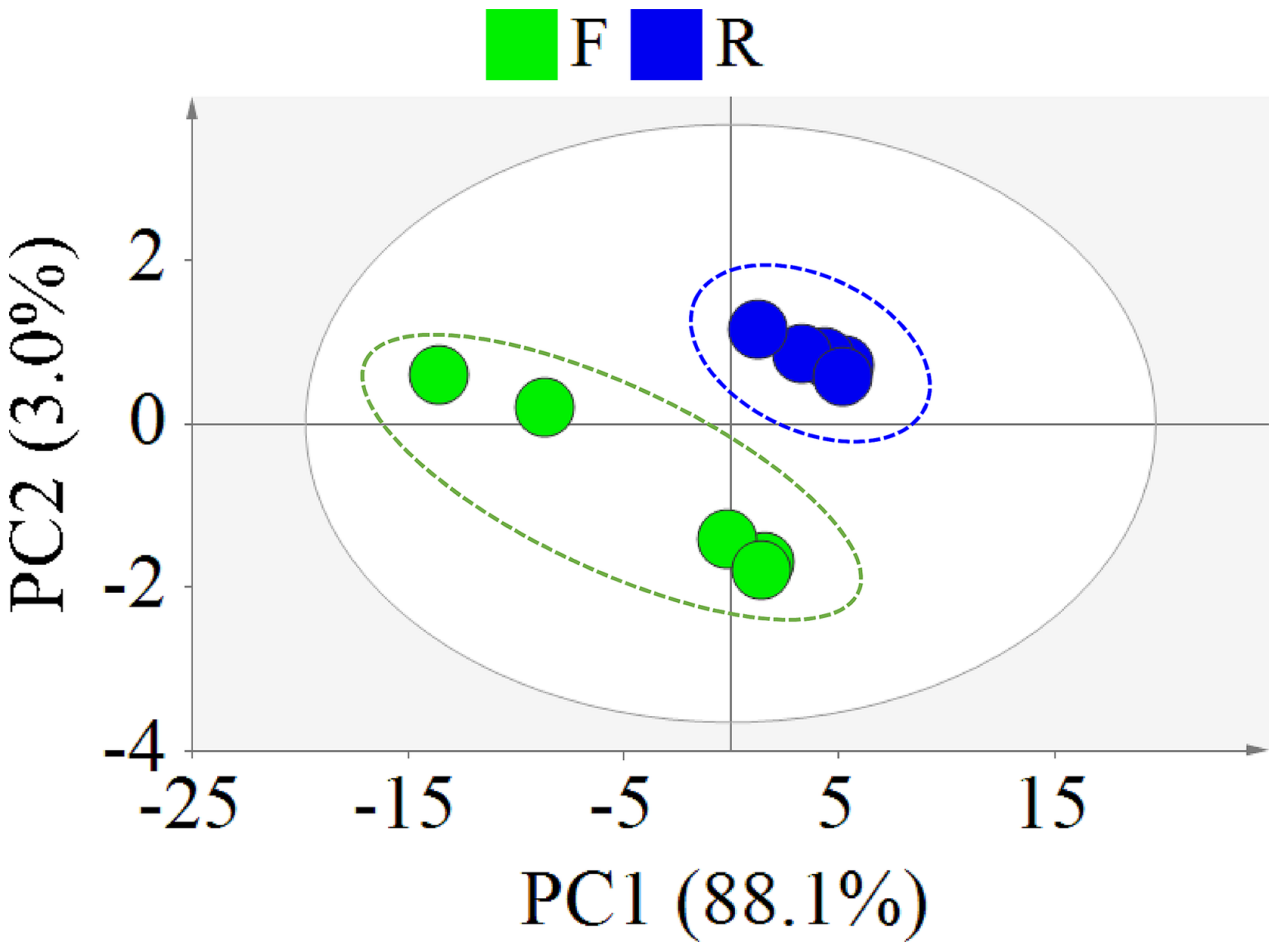
PCA analysis of E-tongue detections.

In addition to functional relevance, the contribution of significantly altered compounds to taste variation was further investigated. Principal component analysis (PCA) was first performed on the taste profiles of raw and stir-fried TS. As shown in Figure 7, a clear separation between raw and stir-fried TS was observed, indicating that stir-frying resulted in significant changes in the taste properties of TS. To further identify compounds potentially associated with taste variation after stir-frying, correlation analysis was conducted between the 83 significantly altered compounds and E-tongue sensor responses. As shown in Table 2, 39 compounds exhibited absolute *ρ* greater than 0.6. Among these compounds, 10 were fatty acids, 7 were FAHFAs, 6 were amino acids and analogues, 4 were PIs, 3 were terpenoids, 2 were DGs, 2 were MGs, 2 were PCs, 1 was a steroid and steroid derivative, and 2 belonged to other compound classes. The diversity of these compound classes suggests that the taste characteristics of TS are unlikely to be determined by a single compound class, but rather arise from the combined contributions of structurally diverse metabolites and lipids. Similar phenomena have been reported in other thermally processed plant-derived foods, in which flavor formation is closely associated with complex interactions among lipids, amino acids, and their thermal degradation products (46, 47).

**Table 2:** Correlation analysis between compounds with significant change and sensory.

Sensors	Compounds	Name	Class	Correlation coefficients/ρ	*p*	Relation
S3	1	Decanoic Acid	Fatty acids	0.68	0.029	positive
S3	3	13-Hotre	Fatty acids	−0.69	0.026	negtive
S6	3	13-Hotre	Fatty acids	−0.64	0.044	negtive
S3	4	Oleic Acid	Fatty acids	−0.67	0.032	negtive
S6	4	Oleic Acid	Fatty acids	−0.64	0.045	negtive
S3	8	9-Hode	Fatty acids	−0.69	0.027	negtive
S6	8	9-Hode	Fatty acids	−0.64	0.048	negtive
S3	10	15,16-Dihydroxyoctadeca-9,12-Dienoic Acid	Fatty acids	−0.68	0.032	negtive
S3	11	Fa 18:4 + 2O	Fatty acids	−0.69	0.027	negtive
S3	12	9-Hotre	Fatty acids	−0.69	0.028	negtive
S3	13	Myristoleic Acid	Fatty acids	−0.67	0.034	negtive
S3	14	Linolenic Acid	Fatty acids	−0.68	0.030	negtive
S3	15	Jasmonic Acid	Fatty acids	−0.68	0.032	negtive
S2	16	2-(14-Methylpentadecanoylamino)-3-Phenylpropanoic Acid	Amino acids	−0.67	0.033	negtive
S3	16	2-(14-Methylpentadecanoylamino)-3-Phenylpropanoic Acid	Amino acids	−0.72	0.019	negtive
S4	16	2-(14-Methylpentadecanoylamino)-3-Phenylpropanoic Acid	Amino acids	−0.65	0.042	negtive
S5	16	2-(14-Methylpentadecanoylamino)-3-Phenylpropanoic Acid	Amino acids	−0.68	0.031	negtive
S6	16	2-(14-Methylpentadecanoylamino)-3-Phenylpropanoic Acid	Amino acids	−0.69	0.026	negtive
S3	17	N-Oleyl-Leucine	Amino acids	−0.66	0.037	negtive
S3	18	Oleyl Sarcosine	Amino acids	−0.67	0.035	negtive
S3	19	N-Fructosyl Phenylalanine	Amino acids	−0.64	0.045	negtive
S3	20	N-Fructosyl Isoleucine	Amino acids	−0.64	0.048	negtive
S3	21	3,9,15-Tribenzyl-6,12,18-Triisopropyl-4,10,16-Trimethyl-1,7,13-Trioxa-4,10,16-Triazacyclooctadecane-2,5,8,11,14,17-Hexone	Amino acids	−0.68	0.031	negtive
S1	22	10-HHTCA	Terpenoids	−0.73	0.016	negtive
S2	22	10-HHTCA	Terpenoids	−0.74	0.014	negtive
S3	22	10-HHTCA	Terpenoids	−0.82	0.004	negtive
S4	22	10-HHTCA	Terpenoids	−0.76	0.011	negtive
S5	22	10-HHTCA	Terpenoids	−0.78	0.008	negtive
S6	22	10-HHTCA	Terpenoids	−0.82	0.004	negtive
S2	23	Chrysanthemyl Alcohol	Terpenoids	−0.65	0.043	negtive
S3	23	Chrysanthemyl Alcohol	Terpenoids	−0.72	0.019	negtive
S4	23	Chrysanthemyl Alcohol	Terpenoids	−0.64	0.045	negtive
S5	23	Chrysanthemyl Alcohol	Terpenoids	−0.67	0.033	negtive
S6	23	Chrysanthemyl Alcohol	Terpenoids	−0.69	0.027	negtive
S2	24	2-Methoxy-5 (6)Epoxy-Tetrahydrocaryophyllene	Terpenoids	−0.63	0.049	negtive
S3	24	2-Methoxy-5 (6)Epoxy-Tetrahydrocaryophyllene	Terpenoids	−0.70	0.026	negtive
S2	25	(R)-4-(3,12-Dihydroxy-10,13-Dimethylhexadecahydro-1H-Cyclopenta[A]Phenanthren-17-Yl)-N-(Pyridin-2-Ylmethyl)Pentanamide	Steroids and steroid derivatives	0.68	0.031	positive
S3	25	(R)-4-(3,12-Dihydroxy-10,13-Dimethylhexadecahydro-1H-Cyclopenta[A]Phenanthren-17-Yl)-N-(Pyridin-2-Ylmethyl)Pentanamide	Steroids and steroid derivatives	0.68	0.030	positive
S3	28	Guanine	Others	−0.66	0.040	negtive
S6	28	Guanine	Others	−0.70	0.024	negtive
S3	29	Kynurenic Acid	Others	−0.66	0.036	negtive
S3	33	FAHFA 27:2; O|FAHFA 18:1/9:0; O	FAHFA	−0.66	0.039	negtive
S1	35	FAHFA 36:6; O|FAHFA 18:3/18:2; O	FAHFA	−0.64	0.044	negtive
S3	35	FAHFA 36:6; O|FAHFA 18:3/18:2; O	FAHFA	−0.67	0.036	negtive
S6	35	FAHFA 36:6; O|FAHFA 18:3/18:2; O	FAHFA	−0.68	0.031	negtive
S3	39	FAHFA 44:4; O|FAHFA 18:3/26:0; O	FAHFA	−0.67	0.035	negtive
S5	39	FAHFA 44:4; O|FAHFA 18:3/26:0; O	FAHFA	−0.65	0.042	negtive
S2	41	FAHFA 26:4; O|FAHFA 18:3/8:0; O	FAHFA	−0.63	0.050	negtive
S3	41	FAHFA 26:4; O|FAHFA 18:3/8:0; O	FAHFA	−0.69	0.026	negtive
S6	41	FAHFA 26:4; O|FAHFA 18:3/8:0; O	FAHFA	−0.65	0.041	negtive
S3	42	FAHFA 42:4; O|FAHFA 18:3/24:0; O	FAHFA	−0.67	0.034	negtive
S2	44	FAHFA 27:4; O|FAHFA 18:3/9:0; O	FAHFA	−0.67	0.035	negtive
S3	44	FAHFA 27:4; O|FAHFA 18:3/9:0; O	FAHFA	−0.72	0.019	negtive
S5	44	FAHFA 27:4; O|FAHFA 18:3/9:0; O	FAHFA	−0.63	0.049	negtive
S6	44	FAHFA 27:4; O|FAHFA 18:3/9:0; O	FAHFA	−0.64	0.046	negtive
S2	45	FAHFA 46:4; O|FAHFA 18:3/28:0; O	FAHFA	−0.65	0.044	negtive
S3	45	FAHFA 46:4; O|FAHFA 18:3/28:0; O	FAHFA	−0.69	0.029	negtive
S6	47	PC 36:2|PC 18:0_18:2	Phosphatidylcholine	0.65	0.043	positive
S3	54	LPC 18:3	Phosphatidylcholine	−0.66	0.037	negtive
S1	59	PI 36:4|PI 18:2_18:2	Phosphatidylinositol	0.68	0.031	positive
S2	59	PI 36:4|PI 18:2_18:2	Phosphatidylinositol	0.83	0.003	positive
S3	59	PI 36:4|PI 18:2_18:2	Phosphatidylinositol	0.84	0.002	positive
S4	59	PI 36:4|PI 18:2_18:2	Phosphatidylinositol	0.82	0.004	positive
S5	59	PI 36:4|PI 18:2_18:2	Phosphatidylinositol	0.82	0.003	positive
S6	59	PI 36:4|PI 18:2_18:2	Phosphatidylinositol	0.80	0.005	positive
S2	61	PI 34:1|PI 16:0_18:1	Phosphatidylinositol	0.64	0.045	positive
S3	61	PI 34:1|PI 16:0_18:1	Phosphatidylinositol	0.70	0.024	positive
S5	61	PI 34:1|PI 16:0_18:1	Phosphatidylinositol	0.65	0.041	positive
S6	61	PI 34:1|PI 16:0_18:1	Phosphatidylinositol	0.63	0.050	positive
S3	62	PI(16:0e/18-HETE)	Phosphatidylinositol	−0.68	0.031	negtive
S3	63	PI 34:2	Phosphatidylinositol	−0.68	0.032	negtive
S2	79	DG 40:2|DG 22:0_18:2	Diglyceride	−0.68	0.031	negtive
S3	79	DG 40:2|DG 22:0_18:2	Diglyceride	−0.75	0.012	negtive
S4	79	DG 40:2|DG 22:0_18:2	Diglyceride	−0.70	0.025	negtive
S5	79	DG 40:2|DG 22:0_18:2	Diglyceride	−0.72	0.018	negtive
S6	79	DG 40:2|DG 22:0_18:2	Diglyceride	−0.66	0.037	negtive
S2	80	DG 37:2|DG 18:1_19:1	Diglyceride	−0.68	0.030	negtive
S3	80	DG 37:2|DG 18:1_19:1	Diglyceride	−0.72	0.020	negtive
S5	80	DG 37:2|DG 18:1_19:1	Diglyceride	−0.63	0.049	negtive
S1	82	Glyceryl linolenate	Monoradylglycerols	−0.76	0.011	negtive
S2	82	Glyceryl linolenate	Monoradylglycerols	−0.65	0.044	negtive
S3	82	Glyceryl linolenate	Monoradylglycerols	−0.75	0.012	negtive
S4	82	Glyceryl linolenate	Monoradylglycerols	−0.69	0.028	negtive
S5	82	Glyceryl linolenate	Monoradylglycerols	−0.71	0.022	negtive
S6	82	Glyceryl linolenate	Monoradylglycerols	−0.78	0.007	negtive
S1	83	MAG	Monoradylglycerols	−0.64	0.047	negtive
S3	83	MAG	Monoradylglycerols	−0.71	0.021	negtive
S5	83	MAG	Monoradylglycerols	−0.65	0.042	negtive
S6	83	MAG	Monoradylglycerols	−0.68	0.030	negtive

Among the 39 correlated compounds, 34 showed negative correlations with taste-related sensor responses, whereas 5 exhibited positive correlations. These findings suggest that the decrease in abundance of multiple compounds after stir-frying may contribute to the observed taste variation in TS. As shown in Table 2, compound 22 (10-hydroxy-1,2,6A,6B,9,9,12A-heptamethyl-2,3,4,5,6,6A,7,8,8A,10,11,12,13,14B-tetradecahydro-1H-picene-4A-carboxylic acid, 10-HHTCA) exhibited the strongest negative correlation (ρ = −0.82) with taste, while compound 59 (PI 36:4|PI 18:2_18:2) showed the strongest positive correlation (ρ = 0.84) with taste. Compound 22 is structurally classified as a triterpenoid. Previous studies have suggested that triterpenoid compounds may contribute to sweet, bitter, and characteristic herbal taste attributes in plant-derived foods (48, 49), implying a potential association between this compound and taste variation in TS. In addition, phospholipids have been reported to indirectly influence flavor perception and mouthfeel properties in food systems (50).

Nevertheless, several limitations should be acknowledged. The present study relied primarily on instrumental E-tongue analysis and statistical correlation, and direct validation through human sensory evaluation was not performed. Therefore, the identified associations should be considered preliminary rather than definitive evidence of sensory contribution. Future studies integrating human sensory panels, quantitative analysis, and flavor recombination experiments may help further clarify the contribution of specific compounds to the sensory properties of TS. Overall, the current findings provide a preliminary compositional basis for understanding the taste variation of TS induced by stir-frying.

## 4 Conclusion

In this study, an integrated LC-HRMS-based metabolomic and lipidomic strategy was applied to systematically characterize the chemical composition of raw and stir-fried TS. A total of 144 metabolites and 295 lipids were identified. Statistical analysis revealed that 29 metabolites and 54 lipids were significantly altered after stir-frying. Notably, most FAs and FAHFAs showed a marked decrease, whereas PCs, PIs and PEs exhibited increased abundance. Based on these differential compounds, molecular docking analysis indicated that 19 upregulated compounds may be potentially associated with constipation-related physiological regulation, suggesting a possible functional implication of the observed compositional changes. In parallel, 39 compounds were tentatively correlated with taste attributes, indicating that flavor variation may be driven by combined contributions from multiple chemical classes. Overall, these results provide a compositional basis linking processing-induced chemical changes with both functional and sensory properties, and highlight potential markers for quality evaluation of TS. Future work may extend this framework by systematically investigating the influence of processing parameters (e.g., temperature and duration) on chemical composition and related properties.

## Data Availability

The original contributions presented in the study are included in the article/Supplementary material, further inquiries can be directed to the corresponding author.

## Footnotes

1
https://pubchem.ncbi.nlm.nih.gov/

2
https://www.uniprot.org/uniprotkb/Q14145/entry#structure

3
http://www.bioinformatics.com.cn/

4
https://www.omicstudio.cn
